# Unexpected emergence from the vegetative state: delayed discovery rather than late recovery of consciousness

**DOI:** 10.1007/s00415-019-09542-3

**Published:** 2019-09-20

**Authors:** Willemijn S. van Erp, Anoek M. L. Aben, Jan C. M. Lavrijsen, Pieter E. Vos, Steven Laureys, Raymond T. C. M. Koopmans

**Affiliations:** 1 Department of Primary and Community Care, Radboud Institute for Health Sciences, Radboud University Medical Center, Radboudumc, ELG 117, Postbus 9101, 6500 HB Nijmegen, The Netherlands; 2 Coma Science Group, GIGA Consciousness, Université de Liège, Liège, Belgium; 3 Department of Neurology, Slingeland Ziekenhuis, Doetinchem, The Netherlands; 4 Joachim en Anna, Center Specialized Geriatric Care, Nijmegen, The Netherlands

**Keywords:** Consciousness disorders (MeSH), Persistent vegetative state (MeSH), Neurological rehabilitation (MeSH)

## Abstract

**Background:**

The vegetative state, also known as the unresponsive wakefulness syndrome, is one of the worst possible outcomes of acquired brain injury and confronts rehabilitation specialists with various challenges. Emergence to (minimal) consciousness is classically considered unlikely beyond 3–6 months after non-traumatic or 12 months after traumatic etiologies. A growing body of evidence suggests that these timeframes are too narrow, but evidence regarding chances of recovery is still limited.

**Objective:**

To identify the moment of recovery of consciousness in documented cases of late emergence from a vegetative state.

**Methods:**

Four cases of apparent late recovery of consciousness, identified within a prospective cohort study, were studied in-depth by analyzing medical, paramedical and nursing files and interviewing the patients’ families about their account of the process of recovery.

**Results:**

All patients were found to have shown signs of consciousness well within the expected time frame (5 weeks–2 months post-ictus). These behaviors, however, went unnoticed or were misinterpreted, leading to a diagnostic delay of several months to over 5 years. Absence of appropriate diagnostics, the use of erroneous terminology, sedative medication but also patient-related factors such as hydrocephalus, language barriers and performance fluctuations are hypothesized to have contributed to the delay.

**Conclusions:**

Delayed recognition of signs of consciousness in patients in a vegetative state may not only lead to suboptimal clinical care, but also to distorted prognostic figures. Discriminating late recovery from the delayed discovery of consciousness, therefore, is vital to both clinical practice and science.

**Electronic supplementary material:**

The online version of this article (10.1007/s00415-019-09542-3) contains supplementary material, which is available to authorized users.

## Introduction

The vegetative state, also known as the unresponsive wakefulness syndrome (VS/UWS) is one of the worst possible outcomes of acquired brain injury [1, 2]. Patients show spontaneous eye opening but no behavioral signs of consciousness. Emergence from VS/UWS is classically considered unlikely beyond 3–6 months after non-traumatic or 12 months after traumatic etiologies [3]. These timeframes, however, are likely inaccurate [4, 5]. The detection of even minimal awareness is of major clinical importance. In contrast to VS/UWS, patients in a minimally conscious state (MCS) [6] show residual sensory and emotional processing, including nociception [7, 8] and have better chances of further recovery [5]. Behaviorally, MCS is characterized by signs of awareness of the self and/ or the environment without functional communication or functional object use [6]. Based on the absence or presence of evidence for language processing, patients are considered respectively to be in MCS− (showing for example visual pursuit or localization of noxious stimuli) or in MCS+ (showing reactions such as inconsistent command following and intentional communication) [9].

Differentiating VS/UWS from MCS unfortunately remains challenging, as reflected by a high rate of misdiagnosis [10]. In this context of diagnostic and prognostic uncertainty, clinicians and families face vital decisions on treatment goals and limitations.

We present four VS/UWS patients in whom signs of consciousness were identified beyond the aforementioned timeframes and propose clinical and scientific recommendations that arise from their stories.

## Methods

Patients were identified within a cohort of 31 patients in VS/UWS at least 1 month post-ictus, founded in the Netherlands in 2012. On-site assessment of level of consciousness (LoC) by a trained clinical researcher (WvE) consisted, at baseline and at each follow-up, of a single Coma Recovery Scale-revised (CRS-R) [11] plus observation of possibly conscious behavior reported by proxies and staff. Findings, including the diagnostic uncertainty associated with this single assessment, were explicitly communicated with the treating physician. We invited families and staff to contact us between follow-up visits if the patient’s reactions changed.

For this case series, we included patients with a CRS-R based VS/UWS diagnosis, in whom the first signs of consciousness were detected during formal follow-up at or after 6 (non-traumatic) or 12 months (traumatic brain injury). Patients’ representatives gave written, informed consent. A medical research ethics committee concluded that no complementary review was needed.

Clinical files were searched for descriptions of possibly conscious behavior. We compared proxies’ accounts of the course of recovery to the chronology of the formal diagnostic process.^1^

## Results (see Table 1; full CRS-R scores available as supplementary material).

**Table 1:** Overview of case findings

Patient	1	2	3	4
Sex, age at incident	M, 49	F, 64	M, 27	M, 61
Fluency in Dutch	No	Yes	Yes	No
Year of incident, etiology	2013, OHCA	2008, SAH	2011, TBI	2014, OHCA
Diagnosis hospital	‘Coma vigil’	‘No LoC diagnosis’	‘Vegetative state’	‘Poor neurological recovery’
Diagnosis nursing home	‘Comatose state’	‘Vegetative state’	‘Vegetative state’	‘Comatose state’
Centrally acting medication with sedative side-effects	None	Valproic acid	Baclofen	Levetiracetam and phenytoin
First documented signs of consciousness (time post-ictus, witness)	Patient repeatedly removes tracheal cannula (2 months post-ictus, staff)	Visual pursuit (7 weeks post-ictus, staff)	Visual pursuit, localization of noxious stimuli (5 weeks post-ictus, staff)	Visual pursuit (6 weeks post-ictus, staff)
First formal recognition of signs of consciousness (time post-ictus, assessor)	Automatic motor behavior (10 months post-ictus, clinical researcher)	Visual pursuit and automatic motor behavior (5.5 years post-ictus, clinical researcher)	Functional communication and object use (16 months post-ictus, clinical researcher)	Automatic motor behavior (8 months post-ictus, clinical researcher)
Somatic events prior to recovery	None identified	Discontinuation of valproic acid and midazolam	Treatment of obstructive hydrocephalus	None identified
Outcome (time post-ictus)	MCS, fully dependent (2 years, 8 months post-ictus)	MCS, fully dependent (7 years, 3 months post-ictus)	Conscious, assisted living, 24-h care. Independent intake of food and drink, verbal communication, mobile (5 years, 4 months)	MCS, fully dependent (1 year, 5 months)

*OHCA* out-of-hospital cardiac arrest, *SAH* subarachnoid haemorrhage, *TBI* traumatic brain injury

### Patient 1

A 49-year-old male, non-Dutch speaking, sustained hypoxic brain damage during an out-of-hospital cardiac arrest in 2013. With a diagnosis of ‘coma vigil’ he was discharged to a regular nursing home, where the physician described his condition as a ‘comatose state’. After two research-related CRS-R assessments corresponding to a diagnosis of VS/UWS, during the third examination, 8 months post-ictus, he showed automatic motor behavior. Medical files revealed that in the first 3 months after the incident, the patient manually removed his tracheal cannula twice and tracked visual stimuli with his eyes. The eventual diagnosis of MCS did not change the patient’s treatment. Two years and 8 months post-ictus, he remains in MCS−, unable to communicate, immobile, and dependent on others for all activities of daily living.

### Patient 2

A 64-year-old female suffered a subarachnoid hemorrhage in 2008 and was transferred to a specialized nursing home without formal LoC diagnosis seven weeks later. Upon admission, the physician concluded she was in a ‘vegetative state’, although she showed ‘some visual tracking’. The patient received valproic acid for epilepsy and increasing doses of midazolam to treat spasticity. Four years and 9 months post-ictus valproic acid was discontinued. Within weeks, the patient’s family noted her smiling in response to the names of loved ones. Her diagnosis did not change, however, until the clinical researcher (WvE) identified visual pursuit and automatic motor behavior, corresponding to MCS at 5.5 years post-ictus. Midazolam was discontinued, but no interventions aimed at further recovery took place. At the time of this study, over 7 years post-ictus, the patient inconsistently follows commands (corresponding to MCS+ ) but is unable to communicate.

### Patient 3

A 27-year-old male sustained extensive traumatic brain injury (diffuse axonal injury, acute subdural hemorrhage and traumatic subarachnoid hemorrhage) during a car accident in 2011. Without explicit LoC diagnosis, 5 weeks later he was admitted to a specialized nursing home with a therapy program aimed at recovery of consciousness. Despite the presence of visual pursuit and localization of noxious stimuli, his physician considered him to be in a ‘vegetative state’. When his reaction pattern deteriorated at 5 months after the injury, all paramedic therapies were discontinued. During research assessment only reflexes were seen. Following transfer to a regular nursing home closer to the patient’s home-town, 8 months after the injury, a brain CT-scan made on the family’s request demonstrated an obstructive hydrocephalus. An intraventricular drain was inserted. The patient recovered command following at 12 months, and functional communication at 16 months post-ictus corresponding to a conscious state. Three years on he lives in a supervised apartment. He eats, drinks and mobilizes independently and communicates verbally.

### Patient 4

A 61-year-old male with limited Dutch proficiency survived an out-of-hospital cardiac arrest in 2014 in a state characterized by the cardiologist as ‘poor neurological recovery’. Upon admission to a regular nursing home, his condition was described as a ‘comatose state’. Six weeks after the incident the patient was noted to follow objects and people with his eyes; at 2 months post-ictus he showed nuanced facial expressions in relation to emotional context. The first formal CRS-R assessment at 5 months post-ictus by the study researcher elicited reflexes only. The family, despite instructing the patient in his native language, could not provoke conscious behavior either. At 8 month follow-up, the patient demonstrated reproducible automatic behavior consistent with MCS−. He was transferred to a specialized nursing home but showed no further improvement.

## Discussion

On first sight, the stories in this case-series might be regarded as unexpected or ‘miraculous’ recoveries worthy of press attention [12]. On closer inspection, all patients showed signs of consciousness within the established prognostic timeframes. Rather than examples of remarkably late recovery of consciousness, these are cases of late discovery of consciousness, remarkable nonetheless [13]. Why was these patients’ potential not recognized sooner?

First, there is the apparent absence of standardized behavioral assessment in daily practice, the inaccurate diagnostic terminology and the misinterpretation of conscious behavior. None of the patients’ medical files or transfer letters mentioned validated scales for LoC determination, let alone repeated CRS-R assessments. Only one patient left the hospital with a formal, though outdated, diagnosis (‘coma vigil’). Upon nursing home admission, two patients were incorrectly labeled as being in a ‘comatose state’, and the other two were considered ‘vegetative’ though both showed signs of consciousness. Those behaviors were, by all accounts, noticed but erroneously interpreted as ‘unconscious reflexes’. When patient 3 subsequently stopped showing signs of consciousness, due to, as it turned out later, the development of hydrocephalus, no expert consultation was sought. Earlier referral for radiological evaluation might have detected this complication sooner.

The second factor concerns the various internal and external influences on patients’ performances. Language barriers (patients 1 and 4), sedative drugs (patient 2) and patient 3′s hydrocephalus are known diagnostic confounders [14–16], as are fluctuations in arousal and awareness [17]. Patient 1, for example, showed only reflexive behavior during assessment while already capable of object manipulation (i.e. removal of his own tracheal cannula) and visual pursuit. Repeated CRS-R measurements might have detected such signs of consciousness earlier. Had it not been for the research project, however, some of the patients’ recoveries might have not been discovered at all.

VS/UWS is a diagnosis per exclusionem: absence of proof is not the same as proof of absence. Only if a stimulus is perceived, processed and gives rise to specific motor behavior we know that someone is conscious [18]. Both input to and output of conscious brain processing may be hampered, thus masking what the patient is perceiving. The detection and, if possible, treatment of epilepsy, low arousal due to sedatives or metabolic impairments, dysphasia, motor impairment, sensory deficit, neglect, attention fluctuations, hydrocephalus, and compensation of language barriers should be regarded as prerequisites for a diagnosis of VS/UWS. On top of that, a formal diagnosis of VS/UWS or MCS warrants a minimum of 5 standardized clinical assessments at different timepoints within a short time interval (e.g. 14 days) [19].

In view of the low prevalence of disorders of consciousness in general [20], and limited specialized care for these conditions even in high-income nations such as the Netherlands [10], it is unreasonable to expect every clinician to be able to discriminate between VS/UWS and MCS. Underestimation of a patient’s awareness, however, may lead to erroneous prognostication, therapeutic nihilism, inadequate clinical and pain management and misinformed end-of-life decisions. Delayed recognition of recovery of consciousness, particularly in long-term care, should also be taken into account during the continuing revision of the prognosis of VS/UWS. A recent publication on late emergence from VS/UWS hypothesized that recovery taking place between formal LoC assessments may go unnoticed [21]. Our case-series proves this to be true. The actual moment of recovery of consciousness can precede its formal recognition by years. Still, this study cannot be seen as proof that late recovery is non-existent.

This study is of modest size and of only partially prospective nature. It is likely that a scientific or clinical context with structural CRS-R based evaluations in place for all patients with prolonged disorders of consciousness would identify late recognition of recovery more often. A mobile team, providing expert-level and evidence-based diagnostic assessment on-site and educating care professionals and families, could minimize future misdiagnoses, diagnostic delays and both scientific and public misconceptions about ‘miracle recoveries’. Bringing the expert to the patient, instead of vice versa, takes away the practical challenges of a clinical transfer, as well as the reduced arousal that often becomes apparent after a patient in VS/UWS reaches hospital. Such a relatively simple innovation would bring patients with prolonged disorders of consciousness closer towards the care and attention they deserve.

## Electronic supplementary material

Below is the link to the electronic supplementary material.

Supplementary file1 (DOCX 16 kb)

## Footnotes

1 Anonymized data will be shared by request from any qualified investigator.
