# Supplementary material for: Unexpected emergence from the vegetative state: delayed discovery rather than late recovery of consciousness
Source: J Neurol. 2019 Sep 20;266(12):3144–9. doi: 10.1007/s00415-019-09542-3 (PMC6851207; doi:10.1007/s00415-019-09542-3)
Supplement: Supplementary file 1 — Supplementary file1 (DOCX 16 kb) [file 415_2019_9542_MOESM1_ESM.docx]

Supplement: CRS-R scores

**Patient 1**

|  | Hospital discharge | Nursing home admission | Research visit 1  4 months post-ictus | Research visit 2  7 months post-ictus | Research visit 3  10 months post-ictus | Research visit 4  2 years, 8 months post-ictus |
| --- | --- | --- | --- | --- | --- | --- |
| Auditory | No CRS-R | No CRS-R | 0 | 0 | 0 | 0 |
| Visual |  |  | 0 | 0 | 0 | 0 |
| Motor |  |  | 1 | 1 | 5 | 5 |
| Oromotor/ verbal |  |  | 1 | 1 | 2 | 2 |
| Communication |  |  | 0 | 0 | 0 | 0 |
| Wakefulness |  |  | 2 | 2 | 2 | 2 |
| Conclusion | ‘Coma vigil’ | ‘Comatose state’ | VS/UWS | VS/UWS | MCS- | MCS- |

**Patient 2**

|  | Hospital discharge | Nursing home admission | Research visit 1  4 years post-ictus | Research visit 2  5 years post-ictus | Research visit 3  5.5 years post-ictus | Research visit 4  7 years post-ictus |
| --- | --- | --- | --- | --- | --- | --- |
| Auditory | No CRS-R | No CRS-R | 1 | 0 | 1 | 3 |
| Visual |  |  | 1 | 1 | 3 | 3 |
| Motor |  |  | 1 | 1 | 5 | 5 |
| Oromotor/ verbal |  |  | 1 | 1 | 2 | 2 |
| Communication |  |  | 0 | 0 | 0 | 0 |
| Wakefulness |  |  | 0 | 2 | 2 | 3 |
| Conclusion | No diagnosis | ‘Vegetative state’ | VS/UWS | VS/UWS | MCS- | MCS+ |

**Patient 3**

|  | Hospital discharge | Nursing home admission | Research visit 1  5 months post-ictus | Research visit 2  16 months post-ictus |
| --- | --- | --- | --- | --- |
| Auditory | No CRS-R | No CRS-R | 1 | 4 |
| Visual |  |  | 1 | 5 |
| Motor |  |  | 1 | 6 |
| Oromotor/ verbal |  |  | 1 | 2 |
| Communication |  |  | 2 | 2 |
| Wakefulness |  |  | 2 | 3 |
| Conclusion | No diagnosis | ‘Vegetative state’ | VS/UWS | Conscious state |

**Patient 4**

|  | Hospital discharge | Nursing home admission | Research visit 1  5 months post-ictus | Research visit 2  8 months post-ictus | Research visit 3  1 year 5 months post-ictus |
| --- | --- | --- | --- | --- | --- |
| Auditory | No CRS-R | No CRS-R | 0 | 0 | 0 |
| Visual |  |  | 0 | 0 | 0 |
| Motor |  |  | 1 | 5 | 5 |
| Oromotor/ verbal |  |  | 1 | 2 | 1 |
| Communication |  |  | 0 | 0 | 0 |
| Wakefulness |  |  | 2 | 1 | 2 |
| Conclusion | ‘Poor neurological recovery’ | ‘Comatose state’ | VS/UWS | MCS- | MCS- |
